# Pro370Leu myocilin mutation in a Chinese pedigree with juvenile-onset open angle glaucoma

**Published:** 2011-06-01

**Authors:** Yan-Tao Wei, Yi-qing Li, Yu-Jing Bai, Mei Wang, Jun-hong Chen, Jian Ge, Ye-Hong Zhuo

**Affiliations:** 1 Visual Science Lab, State Key Laboratory of Ophthalmology, Zhongshan Ophthalmic Center, Sun Yat-sen University, Guangzhou, People’s Republic of China; 2 Department of Ophthalmology, The Second Affiliated Hospital, Sun Yat-sen University, Guangzhou, China; 3 Department of Ophthalmology, Puning People’s Hospital, Guangdong, China

## Abstract

**Purpose:**

To investigate the genotype and phenotype of juvenile-onset open angle glaucoma (JOAG) in a Chinese family (PN pedigree).

**Methods:**

Each family member was comprehensively examined by an experienced ophthalmologist. The clinical characteristics of the family patients with JOAG were documented. Blood samples were obtained from 22 available participants from the PN pedigree. Linkage analysis was performed to identify the possible chromosome loci. The presence of gene mutation was ascertained by polymerase chain reaction amplification and subsequent direct sequencing.

**Results:**

The affected members in the PN pedigree are characterized by early age of onset (mean age at diagnosis is 17 years old), severe clinical presentations, high intraocular pressure (mean IOP of 34.18±2.97 mmHg), and poor response to pharmacological treatment (87.5% of the patients required filtering surgery). The region on chromosome 1 between D1S3464 and D1S1619 was identified in this pedigree by linkage analysis. A Pro370Leu myocilin mutation resulting from a heterozygous C→T transition at the 1,109th nucleotide in exon 3 was detected by gene sequencing. The Pro370Leu mutation co-segregated among all affected individuals of PN pedigree.

**Conclusions:**

The GLC1A Pro370Leu mutation is firmly correlated with a severe POAG phenotype. These data provide clues for the severe disease-causing nature of the Pro370Leu allele. Gene screening may be a useful method for pre-symptom diagnosis and a forewarning to detect the at-risk individuals in familial open-angle glaucoma patients, especially in pedigrees of early-onset.

## Introduction

Glaucoma, one of the leading causes of blindness, is a chronic neurodegenerative disease that affects over 60 million people worldwide by 2010 [1]. Primary open-angle glaucoma (POAG), the most common form, is characterized by painless, progressive, irreversible degeneration of the optic nerve and loss of visual field [2]. Juvenile glaucoma is a relatively rare form of primary open-angle glaucoma that occurs in children and young adults. The exact age of the cut-off between adult-onset and juvenile-onset disease usually falls between 35 and 40 years of age.

Although the underlying etiology is unknown, there is evidence that POAG is a complex heterogeneous disease. According to an epidemiological survey, about 30%–56% of patients with POAG and ocular hypertension (OHT) have a family history, and the incidence in individuals with a first degree relative having glaucoma is about 7–10 times higher than in the general population [3]. Since the first correlated mutation gene (myocilin) was identified in 1997, 14 loci (GLC1A–N) have been linked to POAG [3-5]. Among them, myocilin (*MYOC*), optineurin (*OPTN*), and WD repeat domain 36 (*WDR36*) have been identified as harboring causative mutations [5-7].

*MYOC* consists of three exons and two introns, and encodes a 55–57 kDa protein composed of 504 amino acids. To date, more than 80 missense or nonsense variants of myocilin have been reported in different racial/ethnic populations, with the majority of them being clustered in the conserved olfactomedin domain of exon 3 [8]. Myocilin is a secretary protein and interacts with the components of extracellular matrix in trabecular meshwork. The abnormal function of myocilin has been extensively investigated. Haploinsufficiency does not appear to be the primary disease mechanism of *MYO*C mutations. Current studies demonstrate that mutant myocilin protein is misfolded and accumulated as aggregates in the endoplasmic reticulum, which lead to dysfunction and apoptosis of trabecular meshwork cells.

Herein, an autosomal dominant JOAG family (PN pedigree) residing in Guangdong Province of South China was recruited and subjected to linkage analysis to identify gene mutations. Family history of JOAG was thoroughly recorded, and available family members were examined for glaucoma. Testing included stereo-optic disc photographs, optical coherence tomography, and automated perimetry. The clinical features of the pedigree were assessed and the correlation between the phenotype and the genotype was analyzed.

## Methods

### Subjects

This study was done in accordance with the principles of the Declaration of Helsinki. Informed patient consent and approval by the Hospital Ethics Committee (Zhongshan Ophthalmic Centre, Sun Yat-sen University, Guangzhou, People’s Republic of China) were obtained before initiating the study.

This PN pedigree has three generations and consists of 25 members. The total number of affected individuals was eight. Comprehensive ophthalmologic examinations and general medical history were taken and documented by an experienced doctor (Zhongshan Ophthalmic Centre, Sun Yat-sen University). The protocol included the best-corrected visual acuity using Snellen charts, slit-lamp inspection of the anterior eye, IOP measurement by Goldmann application tonometry, anterior chamber angle evaluation by gonioscopy, and fundus examination including vertical and horizontal optic cup disc ratio (C/D ratio) assessment. All subjects underwent automated visual field examination (tested with Humphrey, SITA fast strategy, program 30–2). The Optical Coherence Tomography (OCT) and color fundus photographs of the disc and macula were tested to aid with assessment of the patients’ visual condition and stage of illness.

The diagnosis of POAG was based on an intraocular pressure (IOP) of 22 mmHg or higher, open angles on gonioscopy, glaucomatous optic disc features, and visual field defects consistent with assessed optic neuropathy. A diagnosis of juvenile-onset open angle glaucoma (JOAG) was given when patients were younger than 35 years at the time POAG was diagnosed. If the person only exhibits an IOP above 21 mmHg (without IOP lowing therapy) in the absence of damage to both the optic nerve and the visual field, that individual will be diagnosed with ocular hypertension (OHT). Topical medication was given to patients with IOPs higher than 21 mmHg. Patients whose IOPs could not be controlled with medicine underwent combined trabeculectomy.

### Linkage analysis

Peripheral blood leukocytes were obtained from all available family members, including eight affected and 14 unaffected individuals. Genomic DNA was isolated from peripheral blood according to standard protocols. Genotyping was performed with three microsatellite markers (D1S3464, D1S210, and D1S1619). DNA samples were subjected to polymerase chain reaction (PCR) amplification using primer sequences and the conditions previously described [9]. Briefly, DNA samples were performed in a 10 μl volume, containing 0.4 mM of each primer, 200 μM dNTPs, 1 U Taq DNA polymerase with a cycling profile of 30 cycles at 94 °C for 30 s, 55 °C for 30 s, and 72 °C for 45 s. The PCR products were separated on 5% denaturing polyacrylamide gel in an Applied Biosystems 377 DNA sequencer (Applied Biosystems, Foster City, CA). Linkage analysis was performed by calculating two-point LOD scores using LINKAGE (ver. 5.1) software suite (provided in the public domain by the Human Genome Mapping Project Resources Center, Cambridge, UK).

### Mutation screening

Primers for exons and exon-intron boundaries were designed for *MYOC* (GenBank AB006686). Primer sequences and their PCR product sizes are presented in our previous paper [9] and presented in Table 1. Genomic DNA (100 ng) was amplified in a GeneAmp PCR System 9700 (Applied Biosystems) with conditions previously described [9]. Briefly, DNA were subjected to a PCR amplification with a protocol of 94 °C for 2 min, 30 cycles at 94 °C for 30 s, 55 °C~58 °C for 30 s, 72 °C for 30 s, and 72 °C for 8 min. The amplified exons were purified and sequenced on an automated DNA sequencer (model 377; Applied Biosystems Inc.). All PCR products were sequenced in both forward and reverse directions.

**Table 1:** The sequences of primers used for mutation screening of *MYOC*.

Primer	Primer sequence 5'→3'	Product length (bp)
E1A	TATTTTCTAAGAATCTTGCTGG	
	TGGATTCATTGGGACTGG	394
E1B	GAAGCCTCACCAAGCCTC	
	GCCTGGTCCAAGGTCAAT	342
E1C	CTGGAGGCCACCAAAGCT	
	AGAAAGGGCAGGCAGGGA	448
E2	CATAGTCAATCCTTGGGC	
	CTGCAGACCTGCTCTGACAA	392
E3A	TTTCTGAATTTACCAGGATG	
	GTCAATGTCCGTGTAGCC	426
E3B	CGGACAGTTCCCGTATTC	
	GCTTGGAGGCTTTTCACA	431
E3C	CAAGACCCTGACCATCCC	
	TGCCCCAAATCACAAGAA	412

## Results

### Clinical phenotype of PN pedigree

**Figure 1:**
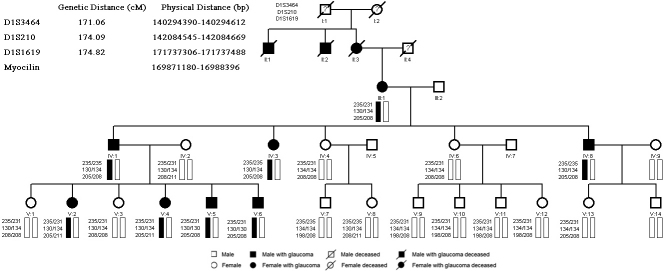
PN pedigree chart with Pro370Leu myocilin mutation.

**Figure 2:**
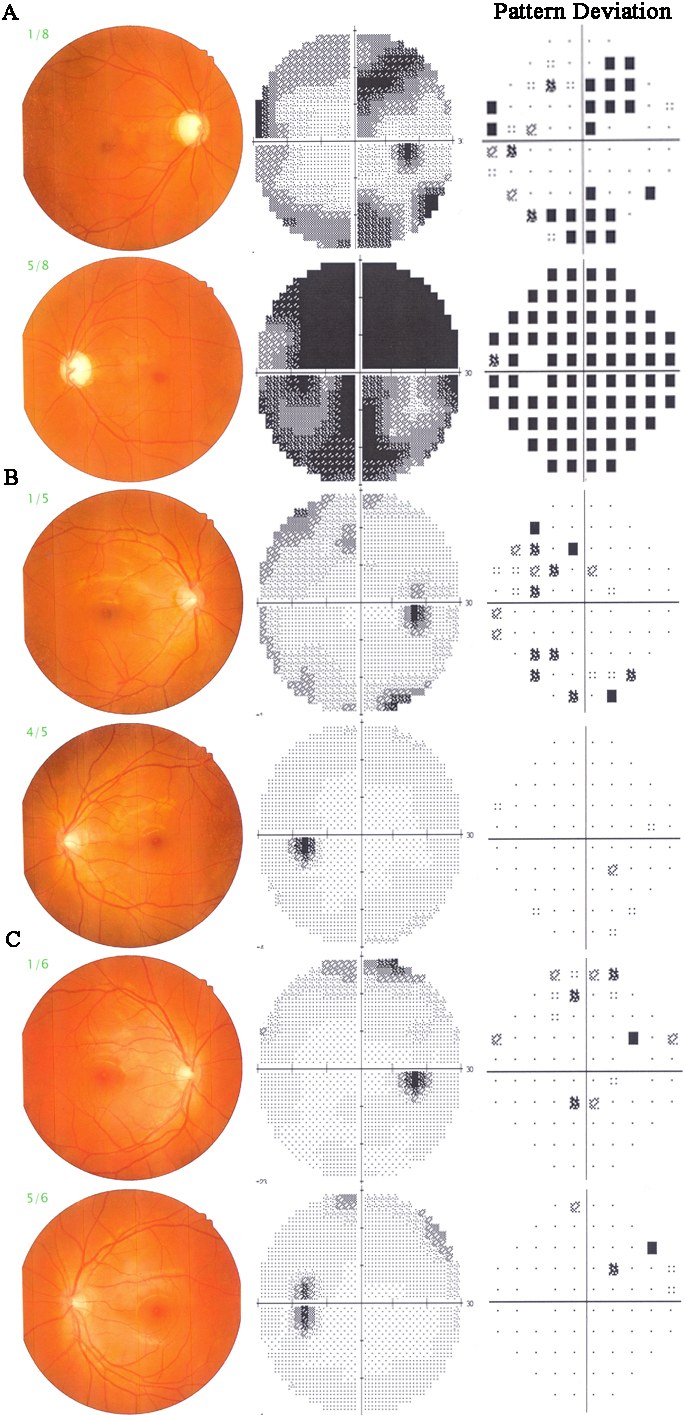
Optic disc photographs and Humphrey 30–2 visual fields in affected patients who carry the Pro370Leu mutation.

According to the distribution of the affected members (Figure 1), the PN pedigree is an autosomal dominant family with eight POAG patients, account for 32% of all family members. Among the affected individuals, four are male and four are female. Patients II1, II2, and II3 were deceased at the time of our study, but their medical records manifest their ocular disease. For the patients with JOAG, the mean age at diagnosis was 17 years (range from 9 to 28 years) and the mean IOP without medication was 34.18±2.97 mmHg (ranged from 29 to 40 mmHg). Examples of optic disc cupping and Humphrey 30–2 visual field defect are given for severe, intermediate, and early cases in Figure 2. Most of the JOAG patients presented poor responsive to anti-glaucoma medications. Seven individuals (87.5%) of the eight mutation carriers studied had undergone glaucoma filtration surgery. All of the surgeries were done at the Zhongshan Ophthalmic Center, Sun Yat-sen University by experienced doctors. After the surgery, all participants obtained their target IOP; none of them needed any follow up anti-glaucoma medications. Only one patient (V6) was still receiving medication during the follow-up. Maximum known IOP, age at diagnosis, and surgical status of the affected individuals are given in Table 2.

**Table 2:** Phenotype characteristic of mutation carriers in PN pedigree.

** **	** **	** **	**BCVA**		**IOP max before Treatment (mmHg)**		**C/D**		**Visual Field Loss**		** **	**Postoperative IOP max (mmHg)**	
**Subject number)**	**Age at diagnosis/Age (years)**	**Gender**	**OD**	**OS**	**OD**	**OS**	**OD**	**OS**	**OD**	**OS**	**Treatment**	**OD**	**OS**
III1	28/68	F	HM	0.15	40	38	1.0	1.0	N/A	Yes	Trab OU	10	11
IV1	20/46	M	0.5	0.4	35	37	1.0	1.0	Yes	Yes	Trab OU	9	12
IV3	26/43	F	0.8	0.8	32	35	0.5	0.7	Yes	Yes	Trab OU	13	12
IV8	18/36	M	0.9	0.8	36	35	0.9	0.8	Yes	Yes	Trab OU	8	9
V2	15/20	F	0.8	0.8	36	33	0.7	0.6	Yes	Yes	Trab OU	9	10
V4	12/16	F	1.0	0.8	35	33	0.6	0.5	Yes	Yes	Trab OU	13	11
V5	10/14	M	1.2	1.5	31	32	0.6	0.6	Yes	No	Trab OU	14	12
V6	9/13	M	1.5	1.5	30	29	0.4	0.5	No	No	Med OU	** **	** **

The Pro370Leu mutation was present in all eight subjects in the table. Seven patients in the table had undergone trabeculectomy surgery to both eyes. Abbreviations in the table are: JOAG; juvenile open-angle glaucoma, BCVA; best-correct visual activity, RE; right eye, LE; left eye, IOP; intraocular pressure, C/D; cup disk ratio, V; vertical, H; horizontal, VF; visual field, F; female, M; male,OU; both eyes, N; normal, IZD; indicated inferior zone defect, NLP; no light perception, LP; light perception, and HM; hand movement, PC; poor cooperation, N/A; unavailable.

### Genotype of PN pedigree

**Figure 3:**
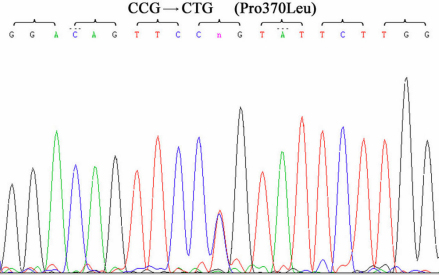
DNA sequence chromatograms of the Pro370Leu MYOC mutation by DNA sequencing in the PN pedigree.

By linkage analysis, two-point LOD scores for D1S1619 and D1S210 were 2.59 (θ=0.0) and 1.63 (θ=0.0), respectively. These markers are located in close vicinity to *MYOC*. Mutation analysis of this gene showed a heterozygous C→T transition at the 1,109th nucleotide in exon 3, resulting in a substitution of proline for leucine (Pro370Leu) (Figure 3). The *MYOC* Pro370Leu mutation was co-segregated among all affected individuals and was not observed in unaffected subjects. Linkage analysis and haplotype analysis demonstrated that all affected individuals were heterozygous for this change (Figure 1).

## Discussion

The myocilin (*MYOC*) gene, the first detected causative gene for glaucoma, is located on chromosome 1q23-q24 and encodes a secreted glycoprotein protein [4,5]. It has been found that mutations in *MYOC* account for more than 10% of dominant juvenile open-angle glaucoma cases and approximately 3% to 4% of unselected adult onset POAG [10,11]. In this study, we described a Chinese family with clinically diagnosed autosomal dominant juvenile-onset open angle glaucoma (JOAG). A heterozygous missense C→T mutation in exon 3 of *MYOC* was found in this family, co-segregating with all glaucoma cases. According to the gene sequence of myocilin, a polar (proline) amino acid was replaced by a hydrophobic (leucine) amino acid due to the C→T transition at nucleotide 1109. No other sequence changes were detected in the entire coding region or splice junctions of *MYOC* in this family.

As previously reported, most of the pedigrees linked to Pro370Leu exhibited an earlier onset and more aggressive glaucoma phenotype [12]. Thus the clinical features of the PN pedigree were thoroughly assessed in our data. The age at diagnosis of affected individuals ranged from 9 to 28 years old (mean 17 years old). Without anti-glaucoma medication, the patients with mutation presented an average IOP of 34.18±2.97 mmHg ranged from 29 to 40 mmHg. Most of the patients required filtering surgery for long-term IOP control because of the poor response to anti-glaucoma medications. Therefore, it is confirmed that the PN pedigree also presents a JOAG phenotype with an early age of onset, rapid progression of the disease, and poor response to medical treatment. The phenotype associated with Pro370Leu correlates well with previous reports in other Chinese JOAG pedigrees [12-16] (Table 3).

**Table 3:** Clinical characteristics of the myocilin mutation in China glaucoma families.

** **	** **	** **	** **	** **	**Age at diagnosis (years)**		**IOP max before treatment (mmHg)**	
**Mutation type**	**Reference**	**Family -base**	**City in China**	**Phenotype**	**Mean (range)**	**Proband**	**Mean (range)**	**Proband**
P370L	Present study	yes	Puning in South China	JOAG	17 (9–28)	21	34.18 (29–40)	45
P370L	[12]	yes	Guangzhou in South China	JOAG	30 (11–35)	20	45.52 (35–56)	37
P370L	[13]	yes	Shanghai in East China	JOAG	22.6 (14–31)	20	41 (30–54)	46
Q337X	[14]	yes	Shanghai in East China	JOAG	24.9 (16–41)	41	30.8 (24–46)	28
C 245Y	[15]	yes	hongkong	JOAG	18 (12–27)	16	26 (24–26)	26
N450Y	[16]	yes	Beijing in North China	JOAG	26.3 (20–31)	20	48.5 (30–56)	N/A

So far, mutations in *MYOC* are reported in POAG patients by multiple research groups in different regions. According to the Myocilin Allele-Specific Glaucoma Phenotype Database, it has been shown that firm genotype–phenotype correlations exist. The most prevalent Gln368Stop mutation may give rise to milder POAG presentation with late onset [17]. The Thr377Met mutation is associated with more severe phenotype of the disease than Gln368Stop mutation [18,19]. The Pro370Leu, Gly246Arg, or Tyr437 His mutations are responsible for the most severe glaucoma phenotypes with early onset [20]. It is remarkable that the Pro370Leu mutation has been found in patients of varying ethnicity, such as French, English, Indian, North American, Japanese, and German populations [21-26]. In Chinese glaucoma patients or pedigrees, 12 *MYOC* mutations have been identified, among which Pro370Leu is the most frequently identified variant. Because it is a prevalent and severe mutant allele both in Chinese and in other ethnicities, further studies are required to clarify the pathogenic roles played by the Pro370Leu mutation in the pathogenesis of POAG with severe phenotype.

The molecular pathway from the glaucoma genotype to the phenotype has not been elucidated. In accordance with previous experiments by different laboratories, most evidence supports the gain of function theory to explain the pathogenesis of myocilin glaucoma. Studies showed that mutant myocilin cannot be secreted in cultured cells; rather, the mutant proteins were misfolded, and accumulated in the endoplasmic reticulum (ER) as insoluble aggregates. This aggregate may induce the procession of ER stress and lead to potential cytotoxicity [27-30]. Furthermore, some important evidence of genotype-phenotype correlation was revealed by Aroca-Aguilar et al. [31]. His experiments revealed that myocilin is proteolytically cleaved at the COOH-terminus of Arg226 by calpain II. Extended study found that the endoproteolytic processing of myocilin was inhibited by different mutations with varying efficiency. For instance, the Pro370Leu led to the highest suppression of endoproteolytic cleavage. Meanwhile, E323K and D380A mutations associated with milder phenotypes produce less intense inhibition. These data provide more clues for the severe disease-causing nature of the Pro370Leu allele.

According to the structure of myocilin, the Pro370Leu transition occurs in the CpG dinucleotide region and also within the highly conserved OLF-domain [32]. It was hypothesized that a single amino acid change in the active region of the protein may result in dramatic changes in the predicted secondary structure. The severe disease-causing characteristics of Pro370Leu indicate that the change in the structure at this position may severely affect the normal processing of myocilin protein in cells, for instance in the folding, conformation, interaction, cleavage, or secretion of the protein. Therefore, these abnormalities might lead to cytotoxicity of the trabecular meshwork cells and in turn impede the pathway of aqueous humor outflow. However, the primary mechanism by which the variant causes JOAG has not been fully unraveled and more events need to be explored.

To date, the frequencies of disease-causing mutations in *MYOC* are similar to those of other ethnic groups (3.86% in Caucasian patients, 3.30% in African patients, and 4.44% in Asian patients) [33]. Considering the low prevalence of *MYOC*-associated glaucoma, it is not feasible to screen whole populations for mutations [34]. Based on our previous research, we performed a prospective cohort study in a large Chinese JOAG family (GZ.1 pedigree) with similar genotype and phenotype to the PN pedigree. During the follow-up of 10 years, all Pro370Leu carriers in the GZ.1 pedigree were diagnosed with open-angle glaucoma [35]. These results provide evidence to prove that DNA screening is a useful method with high specificity and sensitivity for early detection of the at-risk individual in a glaucoma pedigree. Thus, gene screening can be used for pre-symptom diagnosis and forewarning in familial open-angle glaucoma patients, especially in pedigrees with early-onset.
